# Negation Processing in Children with ADHD: The Generic Problem of Using Negation in Instructions

**DOI:** 10.1007/s10936-021-09789-w

**Published:** 2021-08-10

**Authors:** Carolin Dudschig, Barbara Kaup, Jennifer Svaldi, Marco Daniel Gulewitsch

**Affiliations:** Department of Psychology, University of Tübingen, Schleichstr. 4, 72076 Tübingen, Germany

**Keywords:** Negation, Children, ADHD, Imperatives, Ironic Negation Effects

## Abstract

Recent studies have suggested that negation comprehension falls back onto inhibitory brain systems that are also crucial for impulse control and other non-linguistic control domains (Beltran et al., 2018, 2019; de Vega et al., 2016; Liu et al., 2020). Against this backdrop, the present pilot study investigated the use of negation within directional instructions (i.e., “not left”, “now left”, “not right”, “now right”) in children with ADHD and a control group. The results indicate that children in general have a long response delay following negative compared to affirmative instructions. Additionally, there was a tendency for this effect to be more pronounced in the ADHD group. Together, these results suggest that negation processing might indeed demand inhibitory control processes, which are differently available across different subgroups. Thus, the current study provides evidence that using negation in imperatives or instructions is generally rather critical and should be avoided if possible, but that negation use is probably even more problematic in specific clinical populations. Potential implications of these results will be discussed.

## Introduction

A universal phenomenon of human communication is the ability to negate, with negation being a key linguistic operator present across all known natural languages (Horn, 1989). In psychology, negation summarizes a broad range of phenomena, starting from a baby’s crying to communicate to the world that it rejects the current state of affairs (e.g., pain, hunger) up to high-level negation used in the logical sense of denial (e.g., “This is not a banana”) (e.g., Dimroth, 2010; Pea, 1978). Lower-level negations (rejection)—typically volitional and affective in nature (see Dimroth, 2010)—are present very early in our development and the word “no” is among the first words uttered. Nevertheless, there are still many unanswered questions regarding how negation comprehension in the linguistic domain evolves and why it is often considered such an effortful cognitive process. Indeed, negation is often seen as one of the most difficult linguistic structures to integrate during comprehension. Even for healthy adults, negation integration can be a very effortful process (e.g., Deutsch et al., 2009; Dudschig & Kaup, 2018, 2020a; Wirth et al., 2019). The aim of this study was to investigate negation processing in children and to shed light onto the question whether specific clinical diagnoses – specifically ADHD – result in even stronger negation processing issues compared to those reported for healthy control groups. Thus, here we will make the first step towards investigating how negation processing – specifically in imperatives – takes place in children diagnosed with ADHD—compared to a healthy age-matched control group. Investigating negation processing in children diagnosed with ADHD is particularly interesting as ADHD is often associated with specific cognitive impairments in the inhibitory system, a system that has recently been suggested to play a major role in negation comprehension (Beltran et al., 2018, 2019; de Vega et al., 2016).

Negation occurs in various forms and instances and as mentioned above when looking at negation from a development perspective one can see a trajectory from using negation as rejection or refusal (“no”—> don’t want something), negation in its semantic form to express the non-existence (“no elephant”—> there is no elephant) up to negation in its highest logical level in the form of denial (“no, that is not an elephant”) (e.g., Dimroth, 2010; Litowitz, 1998; Pea, 1980). In the present study we investigate a specific from of negation, that is negation comprehension in imperatives with regard to its influence on subsequent behaviour. Why does this type of negation processing seem particularly relevant? We use negation in everyday language, often without thinking about it—despite its increased processing difficulty—and without being aware of the potential consequences for the addressed comprehender. Consider the following sentence: “*Don’t think about a pink elephant*”. This is a well-known example demonstrating the key problem of negation processing. Specifically, communicating a negated information often results in the comprehender cognitively dealing with the to-be-negated information, in this case, the pink elephant. Such unwanted results can be even more critical, if negation is used to stop an undesired behavior. For example, when interacting with children in everyday situations adults would routinely say things such as: “*Don’t cross the street*”, potentially resulting in children performing exactly the behaviour they were instructed to avoid (i.e., crossing the street). Such behavioural effects of negation are also evident in applied clinical settings where behavior modification is desirable, e.g., unhealthy eating habits. Here, negation seems like an ideal operator to express the unwanted behavior and stop it accordingly (e.g., *If*… *then I will not eat chocolate*”) (Adriaanse et al., 2011). However, in such contexts using negation might actually ironically strengthen—rather than weaken—the unwanted behavioural habits (e.g., eating the chocolate bar). Similar issues could be expected in school environments – where a teacher might ask the children “*Don’t look out of the window*” instead of “*Stay focused on the black board*” – with potentially even more severe processing difficulties for specific clinical subgroups. Thus, in the present study we decided to focus on negation processing in setups were directional imperatives (“not left”, “not right” vs. “now left”, “now right”) are used as instructions for a specific behavior and thus on contexts that mimic conditions in which the consequences in everyday life might be particularly relevant.

To date, it is still unclear why exactly negation is so difficult to integrate during comprehension. What are the underlying mechanisms, under what circumstances are these difficulties observable and how can they be overcome? Empirical studies in the language comprehension literature have identified some key indicators of negation integration difficulties and the circumstances under which difficulties mainly occur. First, behavioral studies indicated that negation leads to processing difficulties expressed in longer reading times (e.g., Kaup & Lüdtke, 2006) or generally longer processing times to solve a specific task if it involved a negation operator (e.g., Clark & Chase, 1972, 1974; Just & Carpenter, 1971; Wason, 1961; see Kaup & Dudschig, 2020 for a literature overview). Also error rates following negated statements—for example, imperatives—are typically highly increased compared to affirmative counterparts (e.g., Dudschig & Kaup, 2018, 2020a, b). The specific increase of error rates in behavioral tasks following the use of negation has been of particular interest for studies investigating mental control – and thus gained influence beyond the basic language comprehension literature – and has been named the *ironic effects of negation* (Wegener, 1998). Second, if looking at electrophysiological data, there are a variety of studies showing that negation operators are not instantly integrated into the comprehension process (Dudschig et al., 2018, 2019; Fischler et al., 1983), or in other words that core markers of semantic processing (i.e., the N400) are negation-blind (Palaz et al. 2020). Although some of the negation-related difficulties can be overcome if negation is used in a manner that sentence continuations are predictable (Nieuwland, 2016; Nieuwland, & Kuperberg, 2008), not all types of negation licensing result in full negation integration (see Palaz et al., 2020). Also, for the use of negation in imperatives – which are of particular interest for the present study – there has not been any evidence that licensing avoids the additional processing difficulties.

Ironic effects of negation (e.g., Wegner, 2009; Wegner et al., 1993, 1998) – as briefly introduced in the previous paragraph – offer a striking demonstration of the difficulties associated with the processing of negation and are also among the first negation-related processing issues investigated specifically for certain clinical subgroups. These ironic effects range over a variety of phenomena. Although these effects received reasonable attention in the literature on thought control, mood regulation and memory suppression, they have been way less thoroughly investigated with regard to behavioral control. One of the first studies reporting such effects was presented by Wegener et al. (1998). In their study, participants had to hold a pendulum still. When instructed with a negative command (e.g., “do not move the pendulum sidewards”) participants specifically executed the type of error they were instructed to avoid. The same was not true in a condition where no specific movement was to-be-inhibited. Thus, interestingly, the to-be-avoided instructions typically resulted in more movement types of the to-be-avoided nature than in the neutral case. This research found recent influence in sport psychology (e.g., Berry, 2020), showing that such ironic effects of negation do occur in penalty shooting. For example, if the goalie should be avoided, more shots target the goalie (Binsch et al., 2010a, b). Interestingly, studies have also shown that such ironic effects of negation are even more pronounced in anxious subgroups, in particular, in aiming tasks such as hockey penalty shooting, dart throwing, basketball shooting and serving in a tennis game (Gorgulu, 2019a, b; Oudejans et al., 2013; Woodman et al., 2015). Specifically, anxious participants performed their responses more in the direction of the explicitly to-be-avoided target zones. Thus, these results indicate that it is even more important for vulnerable subgroups to avoid specific types of instructions in coaching.

In the present study we were particularly interested in potential negation processing issues present in children and also specifically in a clinical ADHD subgroup. Despite it being of great relevance how children understand negated imperatives, there have been – to the best of our knowledge – no direct investigations as of yet. Additionally, by means of investigating an ADHD subgroup we aimed at understanding more about the core mechanisms underlying negation comprehension. But what are the exact mechanisms we are targeting? Interestingly, there have been recent suggestions that linguistic negation processing falls back onto rather non-linguistic cognitive mechanisms, specifically the so-called inhibitory control system, often also associated with impulsive control (Beltran et al., 2018, 2019; de Vega et al., 2016). In these studies it was investigated whether negation processing interacts with other tasks (e.g., stop-signal task; go/no-go task) or shows electrophysiological correlates associated with the non-linguistic inhibitory system. Indeed, these studies pointed towards an association between linguistic negation processing and the non-linguistic inhibitory system – whereby a strong interpretation of these experiments would suggest that during negation comprehension the general inhibitory system is actively involved in the comprehension process. The present study focuses on this association, albeit by a different approach. As such, we investigated whether negation processing is particularly demanding for children with ADHD, who are typically characterized by substantial inhibitory control difficulties (e.g., Chmielewski et al., 2019; Gagne et al., 2020). The inhibitory control difficulties observable in children with ADHD are usually investigated across a wide range of tasks (for an overview of tasks see: Sergeant et al., 2002), whereby the most prominent task is the stop-signal task (e.g., Konrad et al., 2000; Nigg, 1999; Schachar & Logan, 1990; Schachar et al., 1995, 2000). In this task there is a primary task where certain responses (e.g., keypresses) to certain stimuli (e.g., visually displayed letters X and O) need to be executed as fast and accurately as possible. Occasionally a stop-signal (e.g., an auditory tone) is presented with a variable delay following the stimulus onset of the primary task and the execution of the primary task needs to be inhibited. Via mathematical models it can then be analyzed how well inhibitory processes are functioning. Across most studies and also in meta-analytic measures the stop-signal task typically shows consistent impairments in ADHD populations with medium effect sizes, however it is less clear how specific these impairments are to this disorder (e.g., Oosterlaan et al., 1998). Combining the recent models regarding negation comprehension – suggesting that negation comprehension relies on inhibitory processes (Beltran et al., 2018, 2019, in press; de Vega et al., 2016) – and the studies from the ADHD literature showing that ADHD is typically associated with inhibitory control deficits – leads to the hypothesis that negation processing might be specifically difficult for an ADHD population.

**Fig. 1:**
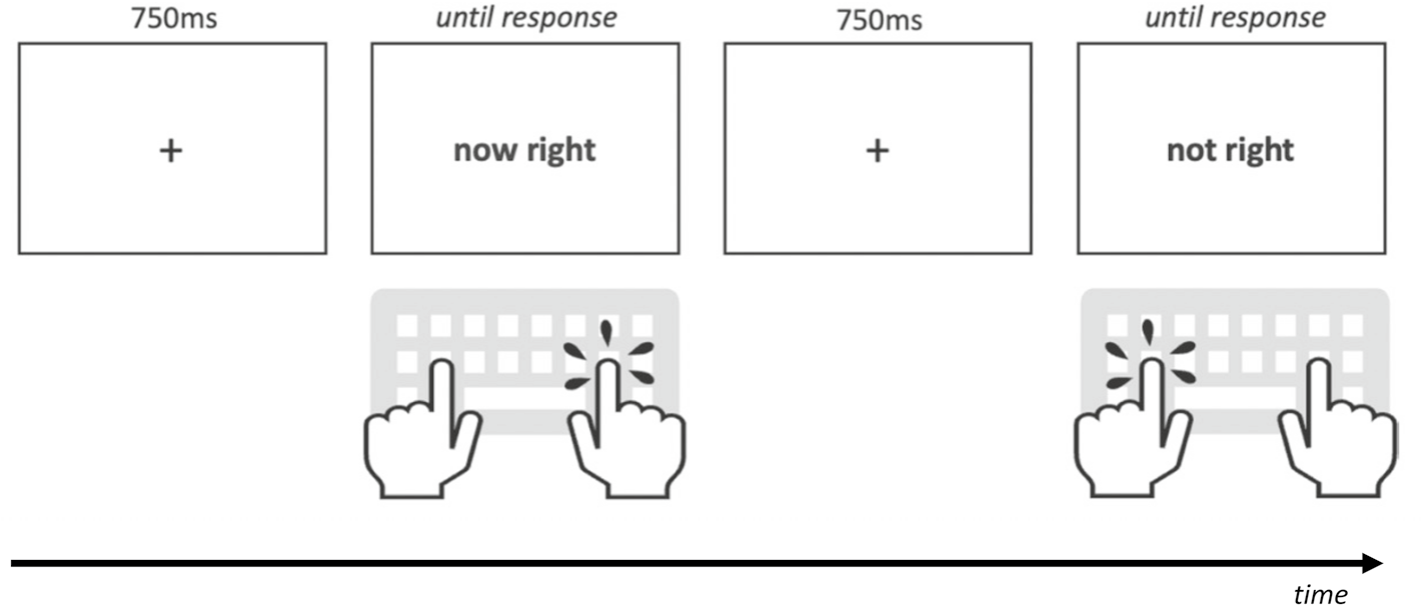
Example trial procedure for two trials, one with the imperative “now right” demanding a right-hand response and one with the imperative “not right” demanding a left hand response

Taken together, the present study aimed at (a) investigating processing of negated imperatives in a child population (b) providing first insights whether an underlying ADHD condition might make negation processing even more difficult. To this end, we used a paradigm—well-established in student populations – that tests how imperatives involving negation are processed (Dudschig & Kaup, 2018, 2020a, b, 2021). In this paradigm, the negation operator is combined with a directional word—resulting in phrases such as “not left”, “now left”, “not right” and “now right”. Participants are required to press the corresponding left/right key (i.e., "not left"—> right key-press). In previous studies with healthy adults the negated items resulted in a large increase in processing time and error rates. These differences persisted when the items were presented in pictorial form (Dudschig & Kaup, 2021). In the present study, we used an identical experimental setup as these previous studies (see Fig. 1). We aimed at investigating how children with and without ADHD deal with negation processing in imperatives and to explore whether there are indications for specific subgroups having greater problems with negation integration.

## Method

### Participants

Overall, 29 participants (17 in control group: *M*_age_ = 11.76, *SD*_age_ = 1.25, 7 female; 12 in ADHD group: *M*_age_ = 11.42 *SD*_age_ = 1.24, 1 female) were recruited by means of university announcements and emails, as well as the waiting list of the adjoint outpatient clinic. Presence or absence of ADHD was based upon the Diagnostic and Statistical Manual of Mental Disorders (American Psychiatric Association [APA], 2013) by a clinical psychologist and psychotherapist. ADHD symptoms were assessed by means of the Conners-3, whose reliability, validity and internal consistency are well established (Lidzba et al. 2013). For the present study, *t*-scores were used to measure severity of ADHD, whereby scores < 60 are considered normal and scores ≧ 65 are considered clinically relevant. Parents gave informed consent and participants were informed and consented to participate before the study. The study was approved via the local university’s ethics board of the Ethics Commission at the Medical Faculty at the Eberhard-Karls University Tübingen (619/2015BO1).

### Stimuli & Procedure

The stimuli and procedure were adapted from previous studies investigating negated imperatives in adults (Dudschig & Kaup, 2018, 2020a, b) with a few adjustments to make the task suitable for children (see Fig. 1). The experiment was programmed in MATLAB using the Psychtoolbox (Brainard, 1997; Pelli, 1997; Kleiner et al. 2007). Each trial started with the 750 ms presentation of a fixation cross in the center of the screen (approx. 1.0 cm × 1.0 cm) in black on a standard light grey background. The fixation cross was followed by the centered presentation of one of the four phrases “jetzt links” (now left), “nicht links” (not left), “jetzt rechts” (now right) and “nicht rechts” (not right) displayed in black (approx. 3.0 cm × 0.8 cm) and participants had to press the according response keys on a standard computer keyboard (left = F-key for “now left” and “not right” vs. right = J-key for “now right” and “not left”). No time-out for the trial duration was implemented, in order to avoid any additional pressure for response time. Each phrase was presented 32 times resulting in an overall number of 128 trials (64 affirmative, 64 negated). The experimental block was preceded by a short practice block of eight trials to familiarize participants with the task. Participants received written instructions (which explained the mapping of the phrases to the correct response keys) on the screen before the practice trials. The experimenter confirmed with the participants that the instructions were comprehended and gave additional verbal instructions if required.

## Results

**Fig. 2:**
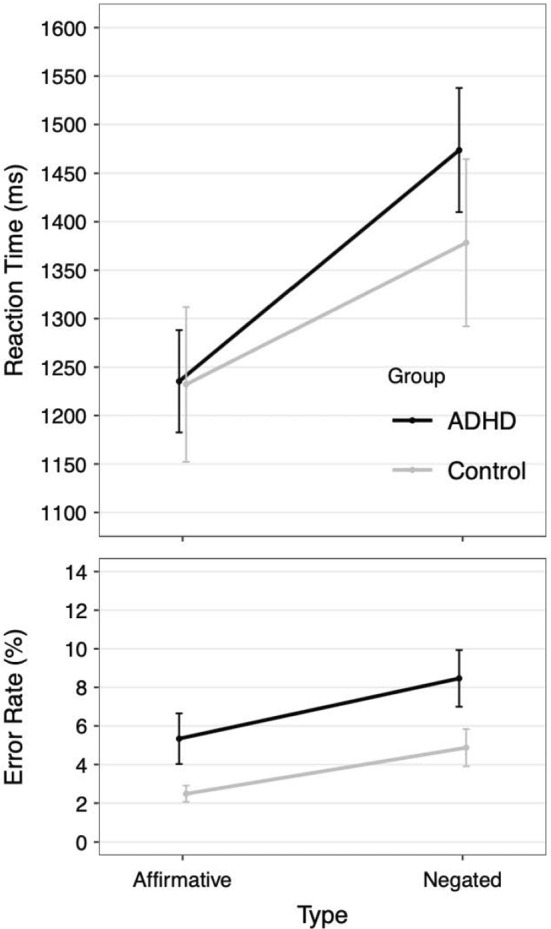
Top plot: Mean reaction times (top) and errors rate (bottom) for the affirmative and negated trials separated for the ADHD and control group. The error-bars represent +—1SEM

The data was analyzed using R (R Core Team, 2017) with one ANOVA for the reaction times (RTs) and one for the error rates using the within-factor polarity (affirmative vs. negated) and the between-factor group (ADHD vs. control). Too fast (< 150 ms; < 0.01%) and too slow (> 3500 ms; < 0.1%) responses were excluded from the subsequent analysis. The ANOVA on the RTs was performed on correct trials only and showed a main effect of polarity, with faster responses in the affirmative (1233 ms) compared to the negated condition (1418 ms), *F*(1, 27) = 94.67, *p* < 0.001. There was no main effect of group in the reaction times (*F* < 1). However, the interaction between group and polarity was significant, *F*(1, 27) = 5.76, *p* = 0.024, which is reflected in the larger increase of the negation effect in the ADHD group (see Fig. 2, top plot).^1^ The ANOVA of the error rates showed again a main effect of polarity with more errors in the negated condition (6.36%) compared to the affirmative condition (3.66%), *F*(1, 27) = 10.57, *p* < 0.01. Additionally, there was a main effect of group, with the ADHD subgroup overall producing more errors (6.90%) than the control group (3.68%). In contrast to the RTs, there was no interaction between polarity and group, *F* < 1. Given the mixed results from the RT and the error rate analyses we also conducted an analysis of the combined measure^2^—the inverse efficiency score (IES; Townsend, & Ashby, 1978, 1983; cf. Bruyer & Brysbaert, 2011). The ANOVA showed again a main effect of polarity, *F*(1,27) = 83.31, *p* < 0.001, but no main effect of group, *F*(1,27) = 0.83, *p* = 0.369. However, the interaction between polarity and group was significant also for the combined measure of RTs and error rates, *F*(1,27) = 5.32, *p* = 0.029.

## Discussion

The present study followed two main aims (a) investigating whether negation comprehension in imperatives results in processing delays in a childhood population, similar to previous studies investigating student populations (b) analyzing the influence of an underlying ADHD condition on negation processing in a first explorative approach. The results showed that in line with previous studies in student populations (e.g., Dudschig & Kaup, 2018, 2020a, b), younger age groups also show a rather large delay in responding following negation. The influence of negation was clearly present both in RTs and error rates, with trials in the negation condition resulting in slower and more error-prone responses. Interestingly, specifically within the RT measurement, the results suggested that these effects are even more pronounced for the ADHD subgroup.

To our knowledge, the present study is the first investigating the processing of imperatives and their behavioral consequences in children. The results clearly indicate that, negation results not only in slower but also more error-prone behavior in children. This also suggests that negation in everyday life – especially when used in imperatives (e.g., "*Don't cross the street!*") – likely prompts avoidable errors or at least slows information processing. The additional influence of comorbid ADHD on negation processing – resulting in increased processing times – is also interesting, as it points to the relevance of an inhibitory processing network for negation integration as proposed recently in the literature (Beltran et al., 2018, 2019; de Vega et al., 2016). One could speculate that this increase in processing time for negation integration is due to the need of non-linguistic inhibitory networks in the processing of negation – processing networks which are typically impaired in ADHD subgroups. Therefore, the ADHD subgroup might need more cognitive effort or generally more time for the recruitment of inhibitory systems that are required for negation integration. It is impossible to disentangle why RTs but not error rates showed an influence of underlying ADHD at the current stage, as RTs and error rates are often treated to reflect the same processing mechanism. We therefore conducted the combined analysis on error rates and RTs which also showed the influence of an underlying ADHD condition on negation processing. Interestingly, the literature regarding ironic effects of negation typically focused on the occurrence of errors and showed that to-be-avoided errors are more likely to occur following a negation instruction. However, given the direct dependencies between error rates and RTs during information processing (e.g., Townsend, & Ashby, 1978, 1983; cf. Bruyer & Brysbaert, 2011), future studies might benefit from taking additional RT analyses into account – which in our study here seem the main indicator regarding fine-grained processing differences between subgroups.

Our conclusion that inhibitory deficits as present in ADHD might impair certain linguistic processes—in the present case, negation integration—should however be considered explorative evidence. First, the sample size was very small (see e.g. Button et al., 2013 for resulting problems). Second, the debate concerning the core cognitive system that is affected within ADHD groups is still ongoing. Beyond deficits in the inhibitory system there have been various other cognitive specificities associated with ADHD, some of them more or less related to inhibitory control processes (e.g., suppression mechanisms, risk-taking; Bauermeister et al., 2007; Matthies et al., 2012, 2014). Thus, other potential modulating factors might explain the results of the present study (see also Banaschewski et al., 2004). However, it is also important to note that many other general factors that we did not control for in the present study (e.g., motivation (Slusarek et al., 2001); attention; decreased reading abilities, etc.) would have probably influenced the affirmation and negation trials in a similar way and therefore at the moment probably do not provide a better explanation for the observed data pattern. Third, although it is typically argued that ADHD deficits result from deficits in the inhibitory system, it is up to date rather unspecified how exactly the inhibitory system is defined, leaving open whether impairment is a general phenomenon or rather specific to motor-related processes (see also Nigg, 2001). Nevertheless, the basic idea that the ADHD population might be particularly interesting for research on negation comprehension – due to deficits in the inhibitory system—is not unjustified. For example, a recent carefully controlled study by Boonstra et al. (2010) on adults with ADHD—who never received medication – tested them across a wide range of paradigms looking into the functioning of various sub-mechanisms of executive functions. That study came to the conclusion that ADHD indeed seems mainly a disorder of the inhibitory system rather than of other types of executive functions (planning, working memory, etc.). Thus, we think the inhibitory network – and populations with disorders related to the inhibitory network – might be of particular interest for future negation research.

Notably, the present study does not enable us to determine the exact processing steps underlying the response delay following negated instructions, another question widely discussed with regard to negation comprehension. With behavioural evidence only, it is often difficult to decide where these comprehension or response slowings originate. In the literature there is an ongoing debate whether negation comprehension takes place in a 1-step or 2-step fashion (Dudschig & Kaup, 2018; Kaup et al. 2006). In other words, this research asks whether during negation comprehension we first represent the to-be-negated information (e.g., *left* in the case of “not left” in the present paradigm) and subsequently the integrated meaning (e.g., *right* in the present paradigm), or whether we can skip the first step and directly result in the final meaning interpretation. This discussion can be seen as being orthogonal to the question regarding the involvement of inhibitory networks during negation processing. Whereas the question regarding the 1- vs. 2-step fashion is particularly focused on whether the to-be-negated information becomes activated during comprehension, the question regarding the involvement of the inhibitory network is focused on the underlying cognitive mechanisms that lead to the final meaning representation. For example, in our view it would be possible that negation processing takes place in a 1-step fashion with the inhibitory system being involved in comprehension, for example by never letting the meaning of the to-be-inhibited information surface into the representational level. During a 2-step comprehension process in contrast, the inhibitory system is involving in pushing under threshold the meaning of the to-be-negated information (i.e. left in our example above) after having first surfaced into the representational level. These are all speculations, as up to date it remains also unclear what is the actual role of the inhibitory system during negation comprehension. However, from previous studies with an adult population using psychophysiological measures we have rather clear insights regarding the processing steps involved in the present experimental setup. Specifically, these studies show that the response slowing is most likely triggered by a 2-step process of negation integration (Dudschig & Kaup, 2018, 2020b): Electrophysiological data—specifically the lateralized readiness potential (Coles & Gratton, 1986)—showed that in negation trials first the incorrect (i.e. ipsilateral to the required response = right motor-cortex for “not left” trials, whereas a correct right hand response requires left contralateral motor-cortex activation) and subsequently the correct motor cortex becomes activated. Therefore, at the current stage we assume that similar 2-step negation integration processes are responsible for the response slowing in the younger participants. Future studies could shed more light on these processes for different subgroups. A recent study in a student population showed that negation processing could be facilitated if stimuli are presented in a pictorial rather than a linguistic format (Dudschig & Kaup, 2021). This might also be a promising approach to investigate in the ADHD population to test whether negation processing can be facilitated by symbol format.

In summary, the current study demonstrates that specific issues regarding negation processing are also pronounced in younger subgroups: Negated imperatives result in longer RTs and increased error rates compared to affirmative counterparts. The present results are a first indication that investigating negation processing in specific subgroups might be worth for two reasons: namely, first for getting a better understanding of the cognitive processes underlying negation processing and second, for finding out whether negation should be used with even more care in specific subgroups. We see the current study as evidence that this is a promising approach for future research investigating the role of non-linguistic cognition for linguistic processes.

## Data Availability

Will be made available upon request.

## Footnotes

1 Given the unequal sample size we decided to conduct an additional analysis. We randomly sampled 12 participants from the control group and ran the ANOVAs using these random samples. Running 1000 permutations resulted in the critical interaction still being significant in the samples (*p* = .049).

2 We thank an anonymous reviewer for this suggestion.
